# A Multi-Scale Dense Perception and Scale-Adaptive Approach for Blueberry Ripeness Detection

**DOI:** 10.3390/foods15071161

**Published:** 2026-03-30

**Authors:** Shutao Guo, Ning Yang, Shanchen Pang

**Affiliations:** 1 Qingdao Institute of Software, College of Computer Science and Technology, China University of Petroleum (East China), Qingdao 266580, China; b22070013@s.upc.edu.cn (S.G.); b22070010@s.upc.edu.cn (N.Y.); 2 State Key Laboratory of Chemical Safety, Qingdao 266580, China; 3 Shandong Key Laboratory of Intelligent Oil & Gas Industrial Software, Qingdao 266580, China

**Keywords:** deep learning, object detection, YOLOv12, fruit ripeness

## Abstract

Accurate blueberry ripeness detection is crucial for intelligent harvesting but is challenged by complex orchard environments involving small, dense fruit clusters. This study proposes BBYOLOv12, an improved YOLOv12 model, to address missed detections and ripeness misjudgments. The method integrates a lightweight RepGhost backbone for efficient multi-scale feature extraction, a modified SimAM attention mechanism to enhance feature capture in dense regions, and an improved WIoU loss function to optimize small object localization. Evaluated on a self-built dataset, BBYOLOv12 achieved a mAP@0.5 of 98.97%, mAP@0.5:0.95 of 83.55%, precision of 97.55%, and recall of 97.27%, outperforming baseline and mainstream lightweight models. The model maintains high accuracy with only 2.36 million parameters and 5.59 GFLOPs, reducing complexity relative to the baseline. A practical Graphical User Interface was also developed for real-time detection and statistical analysis. This research provides an effective technical solution for multi-scale, dense perception tasks in agricultural applications.

## 1. Introduction

Blueberries are a highly popular, high-value small berry, whose health benefits are primarily attributed to their rich anthocyanin content [1,2,3]. Anthocyanins, as a class of flavonoid compounds, are abundantly present in the skin of blueberries [4]. This compound is not only the core component of its health benefits, but also gives the fruit a unique color ranging from red to blue, serving as a natural visual indicator of ripeness. Fruit ripening is a complex physiological process involving a series of biochemical, metabolic, and molecular changes, including alterations in firmness, soluble solids concentration, titratable acidity, and volatile compound distribution. These changes collectively influence the fruit’s appearance, texture, flavor, and aroma [5].

Therefore, harvesting at peak ripeness is crucial to meeting consumers’ highest expectations for blueberry quality, flavor, and potential health benefits [6]. However, accurately assessing blueberry ripeness through visual inspection poses significant challenges. For instance, rabbiteye blueberries and some northern highbush varieties exhibit a prolonged period of uniform pink coloration during the color change phase, with complete blue coloration typically indicating full or near-full ripeness. In contrast, southern highbush blueberries often display mottled patches of green, pink, and blue simultaneously, and even when blue coloration appears, the fruit may not be fully ripe [7]. The complex color variations across varieties and maturity stages inherently limit traditional manual assessment methods, which suffer from inefficiency, strong subjectivity, and inconsistent standards. Against this backdrop, developing an automated intelligent system capable of simulating and surpassing human vision—accurately, non-destructively, and efficiently identifying blueberry fruits and determining their ripeness—holds significant theoretical and practical importance for advancing the precision and intelligent upgrading of the blueberry industry.

Currently, numerous studies have applied deep learning models to fruit detection. Early approaches predominantly employed two-stage detectors for fruit recognition, such as Faster R-CNN and Mask R-CNN [7,8]. Zhu et al. proposed a blueberry canopy fruit detection and recognition technique based on Faster R-CNN, which enhances detection speed and improves the target detection algorithm [9]. Priyadharshini and Dolly validated four distinct methods: Convolutional Neural Network (CNN), Regions with CNN (R-CNN), Fast R-CNN, and Faster R-CNN. Ultimately, Faster R-CNN achieved a high accuracy rate of 98% [10]. Mai et al. integrated the classifier with Faster R-CNN, achieving high accuracy on the small fruit dataset [11]. Wan and Goudos applied Faster R-CNN to a multi-fruit detection method, achieving higher detection accuracy and speed compared to traditional approaches [12]. Although the above methods have achieved high accuracy, their computational complexity makes it difficult to meet real-time requirements.

**Figure 1:**
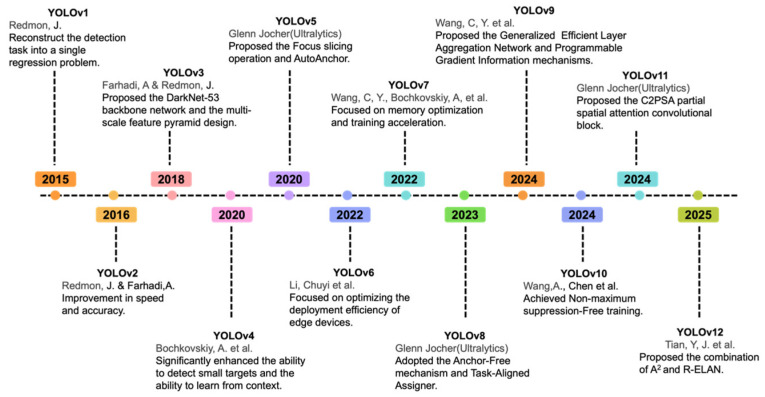
The evolution of the YOLO architecture [13,15,16,17,18,19,20,21,22].

Currently, one-stage detectors, such as YOU ONLY LOOK ONCE (YOLO) and Single Shot Multibox Detector (SSD), are widely adopted due to their high efficiency [13,14]. This algorithm revolutionized the processing workflow of traditional object detection methods by reframing the detection task as a single regression problem. It enables a single convolutional neural network to directly predict both the location and category information of objects from the input image. The YOLO algorithm offers three key advantages: real-time processing, global context awareness, and strong generalization capabilities. However, early YOLO models exhibited certain limitations, notably poor performance in detecting small objects and a restricted number of objects per grid, resulting in low recall rates for densely populated scenes. Figure 1 illustrates the evolution of YOLO. Since its introduction in 2015, YOLO has advanced to YOLOv12, achieving significant breakthroughs in speed and detection accuracy. Today, YOLO algorithms deliver substantial value across industries, including manufacturing, agriculture, remote sensing, and autonomous driving. The model size has been compressed from hundreds of megabytes in v1 to just 7.8 MB in v12, enabling efficient deployment on edge devices.

Singh et al. employed YOLOv8 to detect blueberry ripeness, achieving a mAP@0.5 of 70.8%, precision of 79.7%, recall of 66.3%, and an F1 score of 72.4% for the best model [23]. This indicates that when mainstream general-purpose models are directly applied to a specific target like blueberries, there is still considerable room for improvement in accuracy. Yu et al. proposed a lightweight CNN-based blueberry ripeness detection method, achieving 98.1% accuracy with 2.6 million parameters, offering insights for deployable blueberry ripeness detection systems [24]. Li et al. developed the Yolov7tiny-dGS blueberry ripeness detection model, which improved by 2.5% and 2.8%, respectively, in mAP@0.5 and mAP@0.5:0.95 compared with the original YOLOv7tiny model [25]. Deng et al. employed YOLOv8l and YOLOv9c to detect blueberry ripeness, fruit count, and ripeness percentage, achieving satisfactory results across all metrics. This approach provides valuable insights for blueberry detection in unstructured environments [26]. Although the above-mentioned methods have achieved certain results in detecting the maturity of blueberries, they still face challenges in terms of the universality and robustness of the models. Wang et al. developed the YOLO-BLBE model, integrating the I-MSRCR method to detect blueberry ripeness. This approach provides an effective solution for assessing fruit maturity under various conditions, including different lighting, environmental factors, and natural occlusions [27]. However, there are numerous varieties of blueberries, and the patterns of maturity appearance change among different varieties (such as Rabbit Eye Blueberry and South Highbush Blueberry), showing significant differences. Zhang et al. validated the feasibility of detecting blueberry ripeness using different versions of YOLOv11, with the YOLOv11m model achieving detection accuracies of 90% for ripe blueberries and 81% for unripe blueberries [28]. At the same time, other experimental methods have also been used to assess blueberry ripeness. For example, Yang et al. employed spectral imaging technology to detect blueberry ripeness and leaf classification, achieving classification accuracies of 100%, 100%, 99%, and 98.5% for ripe, semi-ripe, and unripe berries, respectively. Although spectral imaging technology has extremely high precision, its high cost and complex equipment have restricted its large-scale popularization.

All the aforementioned methods demonstrate effective implementation for fruit detection, showcasing the significant potential of deep learning in agricultural applications. However, when these methods are applied to the specific task of blueberry ripeness detection in natural orchard environments, several critical limitations emerge. Blueberry fruits are characterized by their small size, high-density clustering, and frequent occlusion by extensive foliage. These factors collectively pose a dual challenge: conventional detection methods often struggle to accurately distinguish individual fruits in crowded scenes, leading to missed detections and boundary blurring, while simultaneously finding it difficult to maintain high precision and robustness for multi-scale small objects under varying lighting and complex backgrounds. Furthermore, a persistent trade-off exists between achieving high detection accuracy and maintaining a lightweight model architecture suitable for deployment on resource-constrained edge devices. This paper conducts an in-depth investigation into the aforementioned issues, with its innovations summarized in the following four points:

1. Embedding the Density Multi-Scale Similarity-based Attention Module (DMSimAM) into the original YOLOv12 model enhances SimAM’s performance specifically for multi-scale dense object detection.
2. Applying Scale-Aware Wise-IoU (SAWIoU) to YOLOv12 significantly balances detection performance for both large and small objects while improving detection capabilities in dense scenes.
3. Replace the Conv layers in the original YOLOv12 model with the Re-parameterization Ghost (RepGhost) module. This architecture effectively addresses model lightweighting, laying the groundwork for subsequent practical applications.
4. By integrating the BBYOLOv12 (Blueberry YOLOv12) model into a Graphical User Interface (GUI), we developed a comprehensive blueberry ripeness analysis system that combines model loading, data input, real-time detection, and result analysis. This not only demonstrates the practical deployment value of the improved model but also significantly lowers the technical barrier to entry, providing a replicable model for implementing smart agriculture-related research applications.

## 2. Materials and Methods

### 2.1. Dataset and Preprocessing

**Figure 2:**
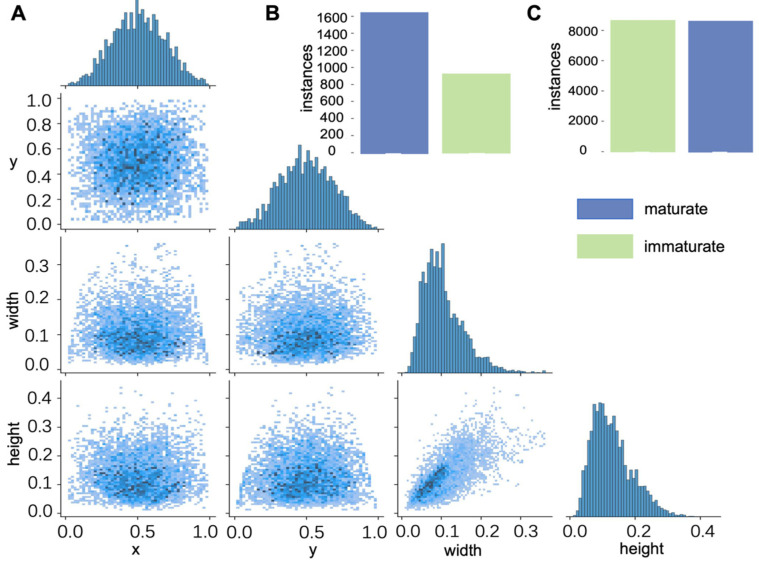
Distribution of dataset features. (**A**) This picture displays the label correlation map of the augmented dataset. (**B**) This picture displays the distribution of the dataset before data augmentation. (**C**) This picture displays the distribution of the dataset after data augmentation.

The dataset used in this study was collected via web scraping (https://image.baidu.com). We conducted a search using keywords related to blueberries and collected pictures of blueberries of different varieties, at different maturity stages, under different lighting conditions, and in different background environments. At the same time, to ensure the quality of the dataset, images that are overly blurred, have too low resolution, or are overexposed or underexposed are excluded. The original image resolution ranges from 640 × 480 to 4032 × 3024 pixels and was captured under various natural lighting conditions (sunny, cloudy, and partially shaded) during the blueberry harvest season (May to August). Camera specifications vary by source, including smartphone cameras and DSLR devices. This dataset covers multiple blueberry varieties and samples at different stages of ripeness, thereby exhibiting a degree of generalizability. The original dataset comprised 192 images, with approximately 1600 mature blueberry instances and 900 unripe blueberry instances (Figure 2B). Given that different varieties exhibit similar visual characteristics during both mature and immature stages, the annotation process uniformly classified blue fruits as mature blueberries and green/red fruits as immature blueberries, ultimately constructing a binary classification dataset. Blue to blue–purple is defined as mature fruit. Specifically, more than 80% of the fruit surface is uniformly blue, possibly with a slight purple tint. Conversely, green to pink fruits are defined as unripe. Specifically, mottled patterns in green, pink, or green and pink, with the blue area being less than 20%. All data were manually annotated using the LabelImg tool. For cases with blurred boundaries (such as fruits with mottled color blocks), annotators are instructed to classify based on the dominant color (50%) and mark the images for expert review. These challenging samples were deliberately retained in the dataset to enhance the model’s robustness to boundary cases.

The maturity of blueberries is mainly visually evaluated by the color of the skin. Reliable model training requires careful color calibration and consistent labeling standards. Although the images come from different network sources and the camera sensors and lighting conditions vary, we applied a color normalization step to reduce the variability between images. Color normalization methods include histogram equalization, white balance correction, and color space standardization, among others. It should be noted that our deep learning method does not require absolute chromaticity calibration because the model learns relative color relationships from a diverse range of training samples.

Given the limited number of target instances and imbalanced class distribution in the original dataset, this paper employs data augmentation strategies to expand the dataset. Before data augmentation, we divided the training set, test set, and validation set in an 8:1:1 ratio. We also use fixed random seeds to ensure repeatability. Hierarchical random division was applied based on approximate instance distribution to maintain the balance of each category in each set. All data augmentation is specifically applied to the training set after the initial dataset is partitioned to ensure that no augmentation images contaminate the validation set or test set. Specific methods include random cropping, rotation, brightness and saturation adjustments, and blurring. After augmentation, the dataset size expanded to 1222 images, with both ripe and unripe blueberry target instances increasing to approximately 9000 each (Figure 2C). This approach effectively mitigated the issues of insufficient sample size and class imbalance while enhancing the model’s robustness and generalization capabilities.

Figure 2A displays the label correlation map of the augmented dataset. This map illustrates how the object detection algorithm models the relationships between labels during training. Each matrix cell represents a label used during model training, with the cell’s color intensity reflecting the correlation between corresponding labels. Darker cells indicate the model has learned a stronger association between these two labels, while lighter cells denote weaker correlations. The colors along the diagonal represent the self-correlation of each label, typically appearing darkest since the model more readily learns relationships with itself. The diagram visually identifies labels exhibiting strong correlations, which is crucial for optimizing training and prediction performance. The four subgraphs of the diagonal in this figure, from top to bottom, respectively, describe the distribution of the x-coordinate of the center point, the y-coordinate of the center point, the width of the bounding box, and the height of the bounding box.

### 2.2. Improved YOLOv12 Network

YOLOv12 is the latest iteration in the YOLO series. The YOLOv12 baseline used in this study is based on the official YOLOv12 implementation released by the original author. Addressing challenges such as quadratic complexity in attention mechanisms, inefficient memory access, and real-time performance requirements, this version integrates three major innovations:

1. Area Attention (A2) provides a concise and efficient solution [29]. Its core idea is to simply divide the two-dimensional feature map into multiple equal-width strip regions along the horizontal or vertical direction. Attention computations are confined within each region rather than across all pixels in the entire image. This approach reduces computational complexity by approximately 50% while retaining a sufficiently large receptive field to capture long-range dependencies, overcoming the limitations of traditional local attention windows. This design achieves an exceptional balance between computational efficiency and model representational power.
2. Residual efficiency layer aggregation network (R-ELAN) [30]. When directly combining traditional CNN feature aggregation modules with attention mechanisms, especially in large-scale models with massive parameters, issues such as weak gradient flow and unstable training often arise. To address this, YOLOv12 designed R-ELAN. It introduces two key improvements to the original structure: First, it adds block-level residual connections with small scaling factors (e.g., 0.01) between the module’s input and output, significantly enhancing gradient flow and stabilizing training for deep networks. Second, it redesigns the feature aggregation path using a more streamlined bottleneck structure, reducing unnecessary computational and memory overhead. R-ELAN ensures efficient and stable feature fusion within complex attention-based architectures.
3. System optimization for real-time detection. FlashAttention technology was introduced, significantly reducing memory overhead and latency during attention computation by optimizing GPU memory I/O access patterns. The positional encoding required in traditional Transformers was removed, replaced by a 7 × 7 separable convolution to implicitly capture spatial information, resulting in a more streamlined model. The MLP ratio in the feedforward network was adjusted to allocate more computational resources to the attention layer itself [22]. These optimizations collectively enable the attention mechanism to operate efficiently within the YOLO framework.

**Figure 3:**
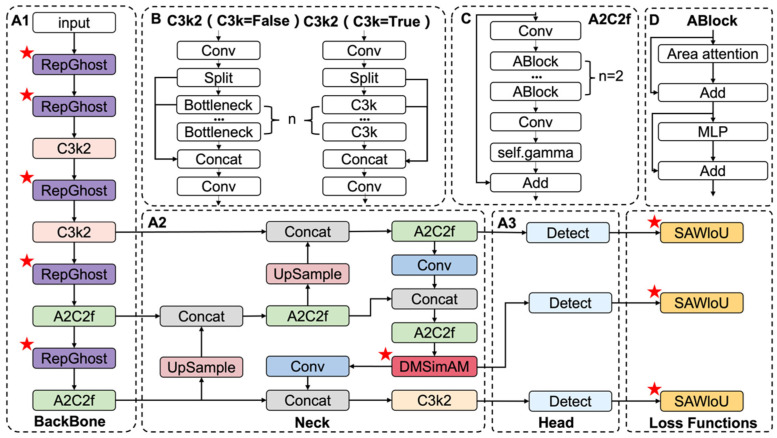
Improved YOLOv12 model. (**A1**) This part is the BackBone component of the YOLOv12 model. (**A2**) This part is the Neck component of the YOLOv12 model. (**A3**) This part is the Head component of the YOLOv12 model. (**B**) C3k2 is one of the modules of the YOLOv12 model. (**C**) A2Cf is one of the modules of the YOLOv12 model. (**D**) ABlock is one of the modules of the YOLOv12 model. The red star indicates that we have replaced and added the YOLOv12 model using an innovative model.

All the modifications proposed in this article (RepGhost, DMSimAM, SAWIoU) are implemented based on the official code base. Figure 3 shows the improved YOLOv12 architecture diagram. A1, A2, and A3 represent the three components of the YOLOv12 network: Backbone, Neck, and Head. B, C, and D depict the network diagrams of the three modules in YOLOv12. This paper introduces improvements based on the characteristics of blueberries, which vary in size and are prone to occlusion. The Neck section incorporates the DMSimAM attention mechanism, optimized for multi-scale dense object detection scenarios. The SAWIoU loss function assigns a higher weight to small object detection, contributing to improved detection of blueberries of varying sizes. Replacing the original Conv module in the BackBone section with RepGhost reduces the model’s parameter count to some extent, providing a solution for subsequent hardware deployment.

### 2.3. RepGhost Model

RepGhost is a core module for building lightweight, hardware-efficient CNNs [31]. It employs structural reparameterization techniques to cleverly fuse the complex multi-branch structure used during training into a single, efficient structure for inference, thereby achieving a better balance between accuracy and speed on mobile and edge devices [32]. Many efficient networks (such as GhostNet) rely on the Concat operation to cheaply increase channel counts and boost capacity. While this operation incurs zero FLOPs cost, it introduces significant latency on real hardware due to complex memory read/write operations.

**Figure 4:**
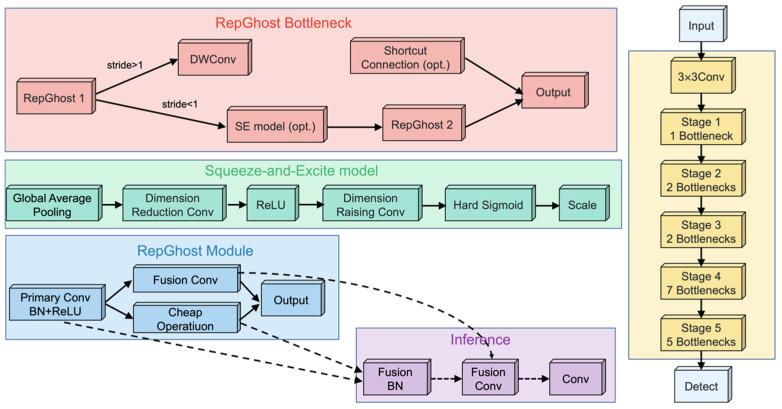
RepGhost structure.

Figure 4 shows the structural diagram of this module. It features two key improvements. The first is replacing concatenation with addition, transforming the feature fusion method from element-wise concatenation to element-wise addition. The addition operation is more hardware-friendly, reducing memory usage and access frequency.

Let the input be $y\epsilon R^{N\times C_{out}\times H\times W}$, output be $x\epsilon R^{N\times C_{in}\times H\times W}$, $\Phi_{i}(x)$, $\forall_{i}=1$, …, s−1 represents other neural network layers applied to x. The process of feature reuse via the Concat operation is described by Equation (1):

(1) $$y=Cat([x,\Phi_{1}(x),\ldots ,\Phi_{s-1}(x)])$$

where Cat represents the Concat operation. It preserves only the existing feature mapping, leaving information processing to the lower layers.

On the other hand, it introduces structural reparameterization. To compensate for the potential decrease in representational power that might result from simply switching to addition, RepGhost borrows from the RepVGG approach by introducing an additional branch during training [33]. These branches enrich the gradient flow, helping the main convolutional kernel learn stronger features. During inference, all branch weights are seamlessly merged into the main convolutional kernel through mathematical transformations. Consequently, the network structure becomes extremely simple and efficient during inference while still benefiting from the performance gains achieved through multi-branch training.

The process of feature reuse through structural parameterization is described by Equation (2):

(2) $$y=Add([x,\Phi_{1}(x),\ldots ,\Phi_{s-1}(x)])=\Phi^{*}(x)$$

where Add represents the Add operation. Unlike the concatenation operation, the addition operation also performs feature fusion. This fusion process occurs in the weight space, introducing no additional inference time, and the resulting architecture is more efficient than one using concatenation.

### 2.4. DMSimAM

SimAM was proposed by Yang, L. et al. in 2021 [34]. SimAM is an attention mechanism based on the local self-similarity of feature maps. It dynamically adjusts the weight of each pixel by calculating the similarity between that pixel and its neighboring pixels within the feature map, thereby enhancing important features and suppressing irrelevant ones.

Extract feature maps $X\epsilon R^{B\times C\times H\times W}$ from the input image using a CNN, where B is the batch size, C is the number of channels, H and W represent the height and width of the feature map, respectively.

For each pixel $x_{i,j}$ in the feature map, where i,j represent the positional indices of the pixel in the feature map, SimAM calculates its similarity to surrounding pixels. For each pixel, calculate the sum of squared differences with all pixels in its neighborhood, then normalize the result. The formula is shown in (3):

(3) $$S_{i,j}=\frac{1}{N}\sum_{k\epsilon \Omega_{i,j}}\|x_{i,j}-{x_{k}\|}_{2}^{2}$$

**Figure 5:**
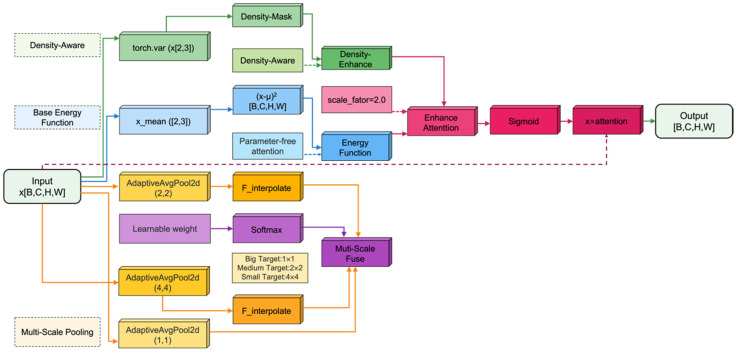
DMSimAM structure.

To achieve greater integration and applicability in blueberry fruit inspection scenarios, this paper systematically enhances the original SimAM attention mechanism (Figure 5). The original method relies on a single-scale energy function, making it difficult to accommodate feature representations of objects of varying sizes. To address this, a three-layer adaptive pooling operation is introduced, constructing large, medium, and small receptive fields, respectively. These operate in parallel to capture multi-scale contextual information, thereby modeling object features in complex scenes more comprehensively. Building upon this, this paper designs a set of learnable scale weight parameters to replace the traditional fixed-weight strategy. These parameters dynamically adjust the contribution ratios of each scale branch through Softmax normalization, enabling the network to adaptively focus on the most discriminative scales based on data distribution. This significantly enhances the model’s flexibility and generalization capability. To address feature confusion in dense object regions, a density-adaptive enhancement module is further embedded. This module identifies high-density areas by calculating spatial variance in feature maps and generates corresponding enhancement masks. It applies an additional 0.5× attention weight to regions classified as dense, amplifying their feature responses and effectively mitigating missed detections and boundary blurring in overlapping objects. Furthermore, to support modern, efficient training paradigms, the entire module explicitly supports mixed-precision training. By incorporating necessary data type conversion and device consistency management, it ensures numerical stability and computational efficiency in FP16/FP32 mixed-precision environments, preventing training anomalies caused by type mismatches. Overall, this enhancement retains SimAM’s lightweight, low-overhead advantages while integrating multi-scale perception, dynamic weight allocation, and density-aware mechanisms. It significantly boosts the model’s adaptability to complex, dense, and multi-scale target scenarios, delivering enhanced robustness for high-precision object detection.

### 2.5. SAWIoU

WIoU was proposed by Tong, Z. et al. in 2023 [35]. Its core idea is to dynamically adjust the contribution of different prediction boxes to the loss, thereby enhancing the model’s generalization capability and robustness [36,37,38,39].

Algorithm 1 presents the pseudocode for SAWIoU. The improved loss function introduces a scale-aware weighting mechanism based on the original WIoU. By applying an exponential decay factor proportional to the object area, smaller targets receive higher weight coefficients in loss calculations, effectively mitigating the issue of large targets dominating gradient updates. Simultaneously, a density-adaptive mechanism is designed to apply additional weighting to small targets and dense regions using a formula, enhancing the model’s learning capability in scenarios with high target overlap. The original WIoU_Scale output is multiplied by the density factor to establish a dual dynamic adjustment strategy, enabling the loss function to synchronously respond to both IoU distribution characteristics and target scale variations. To ensure training stability, clamping operations are introduced during intermediate computations and final output stages. This constrains weights and loss values within reasonable bounds, effectively preventing gradient explosions or training divergence caused by numerical anomalies. This approach is particularly suitable for extended training sessions and mixed-precision training scenarios.

**Algorithm 1.** SAWIoU Loss**Input:** ${box}_{1}=(x_{1},y_{1},w_{1},h_{1}),$ ${box}_{2}=(x_{2},y_{2},w_{2},h_{2})$,ε
1: 
$\;{b1}_{x1}=x_{1}-w_{1}/2,$ ${b1}_{x1}=x_{1}+w_{1}/2,{\;b1}_{y1}=y_{1}-h_{1}/2,$ ${b1}_{y1}=y_{1}+h_{1}/2$

2: 
$inter=max(0,min({b1}_{x2},{b2}_{x2})-max({b1}_{x1},{b2}_{x1}))\times max(0,min({b1}_{y2},{b2}_{y2})-max({b1}_{y1},{b2}_{y1}))$

3: 
$area1=w_{1}\cdot h_{1},area2=w_{2}\cdot h_{2},union=area1+area2-inter+\varepsilon$

4: 
iou=inter/union

5: 
$avg\_area=\sqrt{area1\cdot area2},small\_factor=exp(-avg\_area/8000.0)$

6: 
density_factor=1.0+0.5·small_factor

7: 
wiou_input=clamp(1−iou,ε,1.0)

8: 
$iou\_mean=(1-m)\cdot iou\_mean+m\cdot mean(wiou\_input),m=1-0.5^{1/7000}$

9: 
$\beta =wiou\_input/iou\_mean,\alpha =3\cdot 1.9^{\beta -3},base\_dist=\beta /\alpha$

10: 
enhanced_dist=base_dist·density_factor,enhanced_dist=clamp(enhanced_dist,0.1,3.0)

11: 
return clamp((1−iou)·enhanced_dist,ε−1,1.0)
/*where
$w_{1},h_{1},w_{2},h_{2}$ represent the width and height of the two bounding boxes, respectively. ε is a numerical stability constant typically set to 1 × 10^−7^. clamp() function performs numerical clipping to prevent gradient explosion or underflow.*/

### 2.6. Evaluation Metrics and Environment Configuration

To comprehensively evaluate the performance of the proposed method in object detection tasks, this paper adopts the standard evaluation metrics defined in the Common Objects in Context (COCO) benchmark. The core metrics include average precision (AP), average recall (AR), and their refined variants across different Intersection Over Union (IoU) thresholds and object scales.

Precision is defined as the proportion of positive samples that are correctly predicted as positive by the model, as shown in Formula (4):

(4) $$Precision=\frac{TP}{TP+FP}$$

where True Positive (TP) represents the number of correctly detected targets, while False Positive (FP) indicates the number of false detections.

Recall measures the model’s ability to cover all true targets, as expressed by Formula (5):

(5) $$Recall=\frac{TP}{TP+FN}$$

where False Negative (FN) represents the number of targets missed during inspection.

mAP@0.5 denotes the average precision at an IoU threshold of 0.5, reflecting the model’s detection performance under more lenient localization requirements. It is commonly used for rapid comparisons, with its formula given in (6) and (7). mAP@0.5:0.95 denotes the average AP calculated across 10 IoU thresholds ranging from 0.5 to 0.95. It measures the model’s overall detection capability under varying levels of localization strictness and serves as the most authoritative and commonly used comprehensive accuracy metric.

(6) $$AP=\int_{0}^{1}P(R)dR$$

(7) $$mAP@0.5=\frac{\sum_{i=1}^{n}{AP}_{i}}{n}$$

where n is the number of categories, P and R represent precision and recall, respectively.

Additionally, to accommodate practical deployment requirements, this paper simultaneously considers parameters and GFLOPs to comprehensively evaluate the algorithm’s balance between precision and efficiency.

The experimental setup for this study included both software tools and hardware components. The computational resources comprised an NVIDIA RTX 4090 GPU, an Intel Xeon Gold 5418Y CPU, and 24 GB of RAM. The experiments were conducted using Python 3.10, with CUDA 11.8 utilized for GPU acceleration.

To ensure that all comparison models are evaluated under the same benchmark, we adopted a unified training and inference configuration, as shown in Table 1. All models (YOLOv5, v6, v, v9t, v10n, v11, v12, and the BBYOLOv12 proposed in this paper) use the same hyperparameter settings, including an input image size of 640 × 640 pixels, 100 rounds of training, and a batch size of 64. The optimizer uniformly uses SGD, and the initial learning rate is set to 0.01. In the reasoning stage, the NMS confidence threshold and the IoU threshold are fixed at 0.25 and 0.7, respectively. This standardized experimental protocol ensures that the differences in model performance are entirely due to their respective architectural innovations rather than changes in training configurations.

## 3. Results

### 3.1. Multi-Module Comparison and Synergistic Benefits

To validate the contribution of each improvement module to blueberry ripeness detection, this paper sequentially introduced RepGhost, DMSimAM, and SAWIoU on top of the YOLOv12 baseline model and conducted combined ablation experiments. As shown in Table 2, all metrics were evaluated on our self-built blueberry image dataset. The evaluation metrics are mAP@0.5, mAP@0.5:0.95, precision (P), recall (R), Params, and GFLOPs. In the table, A, B, and C represent the RepGhost, DMSimAM, and SAW IoU modules, respectively. We use “✓” to indicate which modules have been added. Similarly, if no “✓“ is used as the identifier, it means that the module has not been added. The introduction of each of the three innovative modules individually enhances the model’s detection accuracy to varying degrees. This improvement stems from the fact that all three modules are specifically designed to refine and select features characteristic of blueberry fruits. Among them, the RepGhost module helps to reduce the model’s Params and FLOPs. While the number of parameters and computational load are reduced compared to the original YOLOv12 model, indicators such as mAP@0.5, precision, and recall have all been improved. Then, combine the three improved modules in different ways to verify the effectiveness of each module. The experimental results show that the mAP@0.5:0.95 and recall of model ④ have increased by 5.71% and 4.73%, respectively, compared with the baseline, indicating that the lightweight backbone network and dense perceptual attention are highly complementary at the feature extraction level. The mAP@0.5 of model ⑤ reached 98.32%, verifying the synergy between the efficient backbone and scale-sensitive loss in the optimization objective. Although model ⑥ has no improvement in the backbone network, mAP@0.5 still reaches 97.39%, which indicates that the attention and loss function can form an enhancement effect at the feature optimization and gradient guidance levels.

Overall, the improved BBYOLOv12 model shows a favorable trend in all evaluation indicators compared with the original YOLOv12 model. The BBYOLOv12 model achieved 98.97% and 83.55%, respectively, in mAP@0.5 and mAP@0.5:0.95, increasing by 3.11% and 6.63% compared to the baseline, and both precision and recall exceeded 97%. It has been proven that this improved method has a significant effect in detecting the maturity of blueberries.

**Figure 6:**
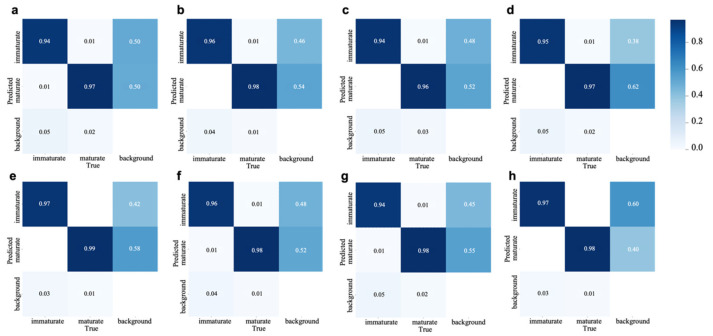
Confusion matrix of the ablation experiment. (**a**) Baseline (**b**) RepGhost (**c**) DMSimAM (**d**) SAWIoU (**e**) RepGhost + DMSimAM (**f**) RepGhost + SAWIoU (**g**) DMSimAM + SAWIoU (**h**) RepGhost + DMSimAM + SAWIoU.

The confusion matrix is the most intuitive and simplest way to evaluate the detection effect of multiple categories. Figure 6 shows the confusion matrices of the eight ablation experiments mentioned above. Each graph corresponds to the classification performance under different experimental Settings. The horizontal axis represents the True category (True), and the vertical axis represents the Predicted category (Predicted). The values indicate the normalized prediction probability. Its color is set from dark to light according to the probability of prediction from high to low; that is, the darker the color, the more accurate the prediction.

When the RepGhost backbone network was introduced, the immature accuracy rate increased to 96%, and the mature accuracy rate increased to 98%, enhancing the feature expression ability and making the fruit boundary clearer (Figure 2b). When DMSimAM was introduced, there were slight fluctuations in the accuracy rates of immature and mature categories (Figure 2c). SAWIoU enhances the gradient of small targets through scale-aware weights, making the model pay more attention to small-sized targets such as immature fruits. Therefore, the accuracy rate of immature fruits increases to 0.95. In terms of the synergy effect of module combination, the performance of introducing RepGhost + SAWIoU is close to that of using RepGhost alone, indicating that the optimization of SAWIoU mainly focuses on the loss function level and does not bring significant gains on the basis of the existing lightweight backbone.

Overall, the detection probability of the main diagonal is significantly higher than that of other positions, indicating that the model has good detection capabilities. The false detection errors of the immature and mature categories are extremely small, and there is almost no phenomenon of misjudging green fruits as ripe ones. Although the background area has a relatively low misjudgment rate, there is still a possibility of being misjudged as a fruit. The detection probabilities of the model for immature and mature fruits have been increased from the original 0.94 and 0.97 to 0.97 and 0.98, respectively, with the accuracy rates for each category improving by 0.03 and 0.01, respectively. This study verified the robustness and practicability of the proposed method in complex agricultural scenarios through systematic comparison.

**Figure 7:**
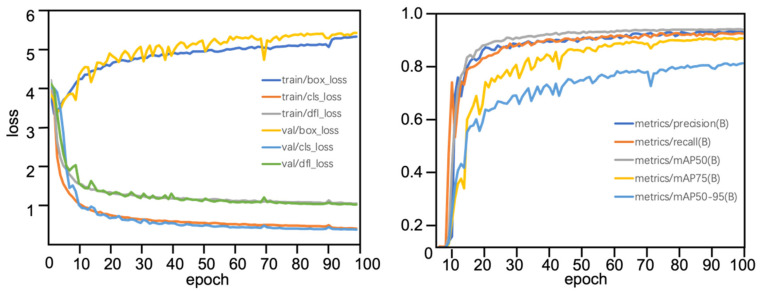
Training dynamics of the BBYOLOv12 model. (**Left**): Evolution of training and validation losses across epochs. (**Right**): Validation performance metrics, including precision, recall, and various mAP scores.

Figure 7 shows the evolution curve of the loss function and the variation curve of the evaluation index of the BBYOLOv12 model during the training process. The left figure shows the changing trends of various loss terms on train and val with training epochs. box_loss represents bounding box regression Loss, cls_loss represents classification loss, and dfl_loss is Distribution Focal Loss. It can be seen from the figure that both cls_loss and dfl_loss converge rapidly and tend to be stable, indicating that the model has proper fitting ability. However, box_loss has shown an upward trend, which seems abnormal from a traditional perspective but has significant explanatory value in modern deep learning. The loss value itself does not have absolute significance; its magnitude depends on the way the loss function is designed. In this paper, the improved loss function may introduce a stronger penalty mechanism, which means imposing higher penalties on wrongly detected targets, enhancing the requirement for bounding box positioning accuracy, introducing category sensitivity weights, and strengthening the target discrimination ability in high-density areas. Therefore, although the total loss has increased, the model is forced to learn more complex feature representations and more robust prediction strategies, thereby enhancing its response ability to key indicators.

The right figure shows the development trends of the key performance metrics: precision, recall, mAP@0.5, mAP@75, and mAP@0.5:0.95 on the validation set as the training process progresses. All indicators showed a trend of rising rapidly at first and then converging slowly. The increase in mAP@0.5:0.95 is significant, and it can still grow steadily in the later stage, indicating that the model has made substantial progress in multi-scale matching ability. The precision and recall curves tend to balance, without showing an extreme bias towards one end, indicating that the model has achieved a significant trade-off between precision and recall. mAP@75 improvement is particularly prominent, meaning that the model can not only detect targets but also locate them more accurately.

### 3.2. Performance Comparison of Existing Models

The BBYOLOv12 model proposed in this study has significantly better comprehensive performance on the self-built dataset than the current mainstream object detection models. To evaluate the statistical reliability of the model performance, we conducted five independent repeated trainings on the BBYOLOv12 model proposed in this paper, using different random seeds (42, 123, 456, 789, 999), with all other training configurations remaining unchanged. The results (mean ± standard deviation) of the five experiments were as follows: mAP@0.5 reached 98.97% ± 0.31%, mAP@0.5:0.95 reached 83.55% ± 0.42%, precision was 97.55% ± 0.28%, and recall was 97.27% ± 0.35%. A smaller standard deviation indicates that the proposed method has stable training convergence and reliable detection performance.

As shown in Table 3, the model achieves 98.97% on the mAP@0.5 metric, which is 3.11 percentage points higher than the existing optimal baseline YOLOv12. More importantly, on the more challenging mAP@0.5:0.95 metric, this method achieved an outstanding result of 83.55%, significantly leading YOLOv12 by 6.63 percentage points, fully demonstrating its breakthrough capabilities in multi-scale IoU matching and precise bounding box positioning. Meanwhile, the model achieved simultaneous improvements in both precision and recall, reaching 97.55% and 97.27%, respectively, far exceeding other comparison models, indicating that it has achieved a considerable balance between reducing false detections and missed detections. At the same time, we also compared the performance of blueberry maturity detection models in the literature. It can be seen that our model shows significant advantages in metrics such as map@0.5, precision, and recall. Moreover, our model also has certain advantages in terms of parameter count and FLOPs, demonstrating the lightweight performance of our model. This model achieved competitive parameter efficiency with 2.36 M parameters, which was lower than the baseline YOLOv12 while maintaining a higher accuracy rate. This indicates that the proposed architectural modification helps improve parameter utilization, but further validation on more diverse datasets is needed to establish a broader generalization statement. This not only verifies the efficiency of the proposed architecture design but also indicates that, through means such as loss function reconstruction, feature fusion mechanism optimization, and improvement of positive and negative sample allocation strategies, this paper has achieved an effective breakthrough where the computational complexity decreases while the accuracy increases. Overall, this result achieves significant synergy between accuracy and efficiency, providing strong support for the actual deployment of lightweight and high-precision detection models. Of course, future work still needs to further examine the robustness and adaptability of the model through cross-dataset generalization experiments and other means. However, the existing results have initially indicated that the proposed method has certain potential and reference value in promoting the exploration of the YOLO series towards higher performance boundaries.

**Figure 8:**
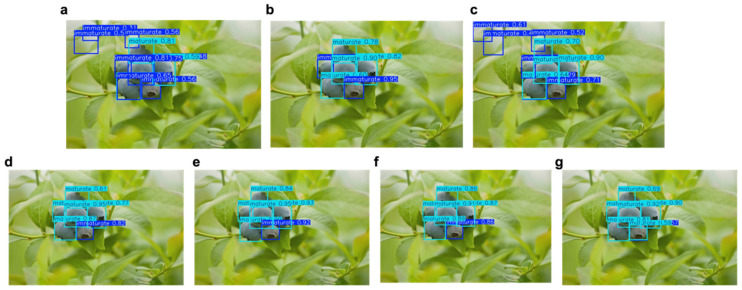
Detection results of the comparative experiment. (**a**) YOLOv5, (**b**) YOLOv6, (**c**) YOLOv8n, (**d**) YOLOv9t, (**e**) YOLOv10n, (**f**) YOLOv11, (**g**) BBYOLOv12.

Figure 8 shows the comparison of detection results of seven mainstream object detection models (YOLOv5, YOLOv6, YOLOv8n, YOLOv9t, YOLOv10n, YOLOv11, and BBYOLOv12) on the same blueberry fruit image. This graph aims to visually present the differences among various models in terms of their ability to recognize small targets, the accuracy of dense area segmentation, and the distribution of confidence in maturity classification. Each subimage contains a bounding box, a category label, and the corresponding confidence score, with the background being the blueberry plant environment under natural light. The YOLOv5 model has encountered issues of missed detections and false detections during detection. As shown in the figure, mature fruits are detected as unripe ones, and the background in the upper left corner is also mistakenly detected as a fruit. It is indicated that YOLOv5 shows a weak detection ability in complex backgrounds and has difficulty accurately distinguishing individuals in high-density areas (Figure 8a). Similarly, YOLOv8n also has the problems of missed detections and false detections as in YOLOv5 (Figure 8c). Although the YOLOv6, YOLOv9, YOLOv10, and YOLOv11 models have relatively solid detection capabilities overall, they all detected the ripe fruits in the lower right corner as unripe fruits. This indicates that these models still have deficiencies in the detection ability of small targets (Figure 8b,d–f). The detection results of the BBYOLOv12 method are relatively complete, and all fruits are correctly framed. The bounding box is closely attached to the edge of the fruit, and it can still accurately locate even in areas blocked by leaves. This indicates that BBYOLOv12 has achieved a high level in terms of detection completeness, classification accuracy, and detection precision, and has reached a high precision in the detection of existing methods, verifying the effectiveness of this improved strategy.

### 3.3. Detection System Based on BBYOLOv12

Traditional manual maturity assessment relies on human visual judgment and essentially has characteristics such as subjectivity and labor intensity, and there are inconsistencies among different workers and at different time points. Fruit detection image processing technology usually relies on manual design, such as color threshold segmentation in a specific color space, combined with morphological operations. These methods have inherent drawbacks, such as being prone to failure when the light changes, and manual parameter adjustment is required for each new variety or new environment. To observe the training effect of this model more intuitively and conveniently, and provide ideas for subsequent research, this paper designs a blueberry maturity detection system based on the BBYOLOv12 model. This Interface is a Graphical User Interface (GUI) designed by PyQt5, which is a computer operating user interface displayed graphically [37]. This interface supports three detection modes: image, video, and real-time camera, and provides real-time statistics and historical management functions for detection data. Our system adopts the advanced YOLOv12 improved model, maintaining high precision even in dense and occluded scenarios. The GUI design takes into account both functional integrity and operational convenience, supporting the storage, query, and export of detection results, and can be used as a front-end tool for the smart farm management system.

**Figure 9:**
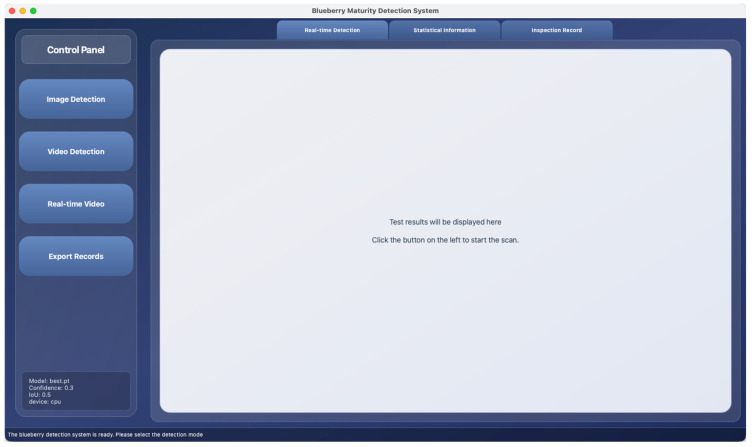
Blueberry maturity detection system main interface.

The main interface of the system adopts a clear left-right partition layout (Figure 9). On the left is a fixed-width control panel, with four main function buttons, namely “Image Detection”, “Video Detection”, “Real-time Video”, and “Export Records”, arranged vertically. Each button features a striking blue-purple gradient design and is equipped with hover highlights and click feedback effects to ensure intuitive operation. The right side is the main display area. The content is organized by the Tab Widget component and is divided into three views: “Real-time Detection”, “Statistical Information”, and “Inspection Record”. Users can switch quickly through the top TAB.

**Figure 10:**
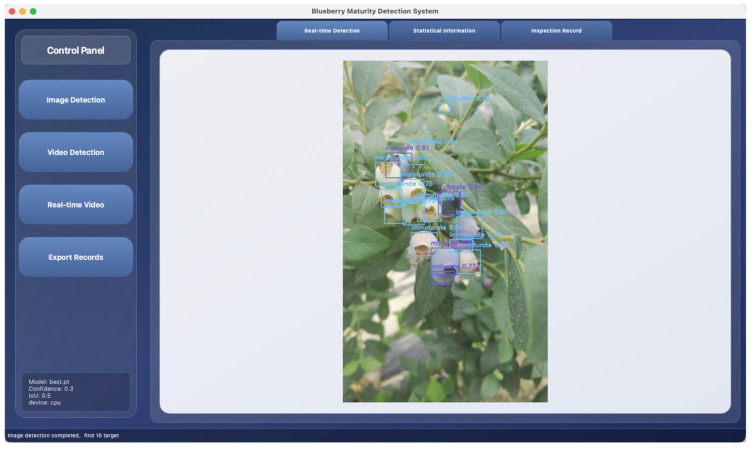
Image detection interface.

The core interaction process directly serves algorithm invocation and result presentation. When the user clicks the “Image Detection” button, the system calls the file selection dialog box, allowing the user to select the local image file. The file formats supported include JPG, PNG, etc. (Figure 10). After selection, the interface passes the image data to the loaded BBYOLOv12 model for inference. After the detection is completed, the bounding box coordinates, category labels, and confidence levels returned by the algorithm are used for visualization on the original image. Among them, the immature category is marked with a light blue rectangular box, the mature category is marked with a purple-blue box, and the Chinese category name and confidence level value are displayed simultaneously above the box. The processed image, after format conversion and adaptive scaling, is displayed in real time in the central area of the “Real-time Detection” TAB. For video and real-time camera modes, the system continuously captures frames through the QTimer timer and performs the aforementioned detection and drawing processes, achieving real-time analysis and result superposition of dynamic video streams.

**Figure 11:**
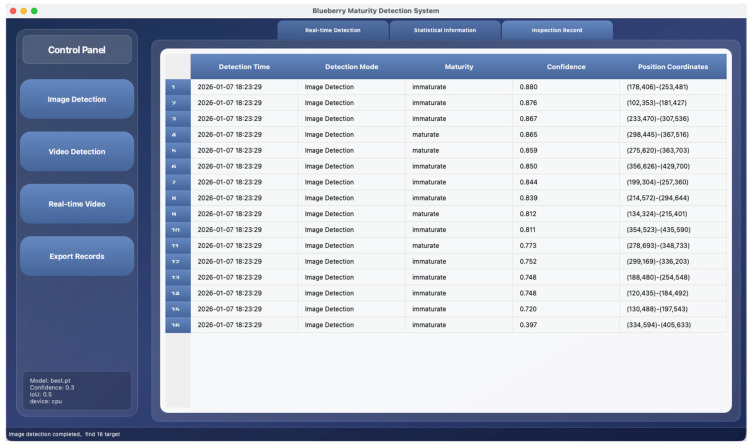
Inception record interface.

To quantitatively display the test results, the system has designed a corresponding data panel (Figure 11). The “Statistical Information” TAB is embedded with a table that dynamically updates and displays the quantity statistics of various types of blueberries in the current test results, facilitating users to quickly grasp the overall maturity distribution. All the results of a single detection, including detection time, detection mode, target category, confidence level, and bounding box coordinates, are automatically recorded and listed in the table on the “Detection Record” TAB. Users can click the “Export Record” button to export all historical records as a structured CSV file with one click for further analysis and archiving.

The interaction with the YOLO model is encapsulated at the bottom layer of the interface through the Detector class, including model loading, parameter setting (confidence threshold, IoU threshold), and inference calls. All detection tasks are processed in a unified function, ensuring the decoupling and efficient collaboration between the interface logic and algorithm modules. The status bar continuously displays the current status of the system, such as the model loading progress, detection mode, and result summary, providing users with clear process feedback. This interface design effectively connects algorithmic capabilities with user operations, encapsulating the object detection function into an intuitive, efficient, and fully functional desktop application.

## 4. Discussion

This study proposes a blueberry maturity detection method based on the improved YOLOv12 model. By introducing the RepGhost backbone network, DMSimAM attention mechanism, and SAWIoU loss function, the accuracy and efficiency of blueberry fruit maturity discrimination have been significantly enhanced. The ablation experiments show that these improved modules not only enhance the model performance on their own but also produce a synergistic effect when used in combination, further optimizing the model’s performance. Specifically, RepGhost enhances the feature extraction capability, especially in handling multi-scale targets; DMSimAM effectively enhances the accuracy of target separation in dense areas. SAWIoU, on the other hand, improved the positioning accuracy of small targets by adjusting the loss weights of targets of different sizes [38].

Comparative experiments show that the BBYOLOv12 model achieves better classification accuracy, positioning precision, and confidence stability in the blueberry maturity detection task compared with other mainstream YOLO variants. Especially when dealing with complex backgrounds, overlapping fruits, and weak texture samples, BBYOLOv12 demonstrates stronger robustness, providing strong technical support for intelligent harvesting in the agricultural field. The proposed GUI system based on the BBYOLOv12 model has multiple practical application values in blueberry production and research. The lightweight architecture makes BBYOLOv12 suitable for deployment on agricultural robots with limited computing resources. The statistical analysis function of the GUI system can automatically count and classify fruits according to their maturity stages. Its intuitive interface and real-time visualization function make this system of great significance for agricultural extension services and agricultural education. The BBYOLOv12 blueberry maturity detection system proposed in this study has certain economic value. This system can effectively reduce labor costs, and automated inspection can reduce reliance on skilled workers, supporting night operations and robot picking. Secondly, the system can reduce harvesting losses, ensuring that mature fruits are picked in a timely manner and unripe fruits are not mistakenly picked. In addition, the system is based on standard RGB images, and its hardware cost is much lower than that of spectral imaging equipment (the price can vary by tens of times), making precision agriculture technology affordable for small and medium-sized farms as well. Finally, the statistical data automatically generated by the system provides a basis for market decisions and enhances consumer trust.

However, the test results showed that a certain proportion of the background was misjudged as fruit. This might be caused by insufficient background diversity in the training set. In the future, it can be addressed by expanding the dataset or introducing background prior information [39]. The lighting conditions of the dataset in this paper are relatively stable. However, under natural conditions, changes in light intensity and direction may affect the generalization ability of the model [34]. Although the dataset has been enhanced in terms of brightness, saturation, etc., it still cannot fully simulate the lighting under natural conditions. Subsequent research may consider adopting adaptive luminance compensation algorithms or conducting tests under more diverse lighting conditions to verify the robustness of the model [35]. Although the model proposed in this paper is superior to most existing algorithms in terms of computational efficiency, compatibility with hardware devices and energy consumption issues still need to be considered in practical applications [36]. For this purpose, more efficient inference engines or edge computing solutions can be considered to ensure that the model can run efficiently on mobile devices [37]. The generalization ability of the model has not been fully verified in cross-dataset scenarios. Although the dataset used in this study covers multiple varieties and different lighting conditions, it is still limited to publicly available images on the Internet. In the future, tests will need to be conducted on blueberry images collected in real fields from different regions, varieties, and growth cycles to comprehensively evaluate the robustness of the model. Although we conducted a multi-random seed stability analysis on BBYOLOv12, due to the limitation of computing resources, we were unable to perform statistical validation on the same scale for all comparison models. Future research should introduce more rigorous statistical testing methods, such as confidence interval analysis and hypothesis testing, to further enhance the persuasiveness of the conclusion.

This study provides an effective solution for blueberry maturity detection and also points out several potential development directions for related fields. In the future, RGB images can be combined with other sensing technologies, such as near-infrared imaging, to utilize complementary information and enhance the accuracy of maturity detection [38]. Meanwhile, systems that can automatically update parameters based on newly collected data can be developed to address the challenges brought about by factors such as differences in crop varieties and changes in growth cycles [39]. To ensure the universality and transferability of this model, the current framework can be extended to similar evaluations of other fruits or crops in the future.

## 5. Conclusions

This paper explores the blueberry maturity detection task based on the YOLOv12 model and constructs the BBYOLOv12 model that takes into account accuracy, efficiency, and robustness. By introducing the RepGhost backbone network, DMSimAM, and SAWIoU loss functions, practical challenges such as small target recognition, dense fruit occlusion, and strong background interference have been effectively addressed. The BBYOLOv12 model achieved mAP@0.5, mAP@0.5:0.95, precision, and recall of 98.97%, 83.55%, 97.55%, and 97.27%, respectively, on the self-built dataset, significantly outperforming the current mainstream YOLO models. Meanwhile, the method we proposed verified the influence of the independent action and collaborative mechanism of multiple modules through ablation experiments. Compared with the baseline model, the evaluation indicators such as mAP@0.5, precision, and recall all improved by more than 3%. While ensuring the detection accuracy, the number of parameters and GFLOPS were reduced by 0.16 M and 0.39 GFLOPS, respectively. This paper also builds a Graphical User Interface based on the improved algorithm BBYOLOv12, which can realize multi-terminal detection of images, videos, and cameras. Meanwhile, the content of the detection report includes information such as detection time, detection model, and number statistics, and can be queried and exported to the local machine. Despite this, there is still room for improvement in the model’s ability to suppress complex backgrounds. Future work will focus on introducing background modeling strategies, expanding multi-spectral information fusion, and exploring cross-variety and cross-regional generalization capabilities to promote the application of this technology in large-scale agriculture.

## Figures and Tables

**Table 1:** Unified training configuration for all models.

Parameter	Setting
Input image size	640 × 640 pixels
Epochs	100
NMS confidence threshold	0.25
NMS IoU threshold	0.7
Batch size	64
Optimizer	SGD
Learning rate	0.01

**Table 2:** Ablation experiment of the improved method of the YOLOv12 model.

Model	A	B	C	map@0.5	map@0.5:0.95	P (%)	R (%)	Params	FLOP (G)
YOLOv12				95.86	76.92	93.95	92.08	2,520,054	5.98
①	✓			97.4	78.83	94.67	95.1	2,361,730	5.59
②		✓		97.08	77.68	93.89	93.89	2,520,054	5.98
③			✓	96.79	77.99	94.32	94.86	2,520,054	5.98
④	✓	✓		97.95	82.63	95.27	96.81	2,361,730	5.59
⑤	✓		✓	98.32	80.01	95.25	95.92	2,361,730	5.59
⑥		✓	✓	97.39	78.49	94.94	94.42	2,520,054	5.98
⑦	✓	✓	✓	98.97	83.55	97.55	97.27	2,361,730	5.59

Numbers ① to ⑦ in the table represent 7 ablation experiments, which are, respectively. ① RepGhost. ② DMSimAM ③ SAWIoU ④ RepGhost + DMSimAM ⑤ RepGhost + SAWIoU ⑥ DMSimAM + SAWIoU ⑦ RepGhost + DMSimAM + SAWIoU. And A, B, and C represent the RepGhost, DMSimAM, and SAW IoU modules, respectively. P, R, Params, and FLOPS represent the precision, recall, parameters, and Floating Point Operations Per Second, respectively.

**Table 3:** Performance comparison between BBYOLOv12 and other mainstream detection models.

Model	mAP@0.5	mAP@0.5:0.95	Precision (%)	Recall (%)	Params	FLOPs (G)
YOLOv12	95.86	76.92	93.95	92.08	2,520,054	5.98
YOLOv11	95.24	74.4	92.32	92.21	2,590,230	6.44
YOLOv10n	93.34	73.46	91.13	88.33	2,707,820	8.4
YOLOv5	94.77	73.94	92.86	91.71	2,508,854	7.18
YOLOv6	93.56	70.73	92.57	87.83	4,238,342	11.87
YOLOv8n	93.09	68.79	90.38	88.8	3,011,238	8.2
YOLOv9t	95.2	75.02	93.44	92.27	2,005,798	7.85
BluberryNet [24]	98.1	97.5	/	95.5	2.6 (m)	7.2
YOLOv7tiny-DGS [25]	92.9	74.2	/	/	6.2 (m)	/
YOLO-BLBE [27]	98.14	/	93.72	97.56	/	/
ours	98.97	83.55	97.55	97.27	2,361,730	5.59

## Data Availability

Due to the composite nature of the dataset (images are collected from multiple public libraries with different licenses), we cannot directly redistribute the compiled dataset. If researchers have such a request, we will provide a complete list of source URLs as well as standard COCO format annotation files.

## Abbreviations

The following abbreviations are used in this manuscript:

CNN	Convolutional Neural Network
YOLO	YOU ONLY LOOK ONCE
SSD	Single Shot Multibox Detector
WIoU	Wise-IoU
RepGhost	Re-parameterization Ghost
GUI	Graphical User Interface
A2	Area Attention
R-ELAN	Residual efficiency layer aggregation network
